# Correction: Of Lice and Math: Using Models to Understand and Control Populations of Head Lice

**DOI:** 10.1371/annotation/c6af2423-f7bd-4b2d-b03a-75019c21e7fe

**Published:** 2011-08-05

**Authors:** Mara Fabiana Laguna, Sebastián Risau-Gusman

The first author's name is misspelled. The correct first author name is: María Fabiana Laguna 

