# Supplementary material for: Of Lice and Math: Using Models to Understand and Control Populations of Head Lice
Source: PLoS One. 2011 Jul 20;6(7):e21848. doi: 10.1371/journal.pone.0021848 (PMC3140471; doi:10.1371/journal.pone.0021848)
Supplement: Text S1 — In this supplement we provide complementary material organized in two sections. In Section A, the details of the two sets of data used to build our models are given. We include additional tables built from the original data sets. We also give a brief description of the algorithm we use to perform the numerical simulations shown in the paper. In Section B we provide figures and tables built with the ES data, analogous to the ones in the main text. To make the comparison easier, figures and tables of this section have the same number as their analogue in the main manuscript, but preceded by a letter B. (PDF) [file pone.0021848.s001.pdf]

Text S1: Supporting information for 'Of lice and math: using models to understand and control populations of head lice'

María Fabiana Laguna, Sebastián Risau-Gusman

Section A: Data and Algorithm

In this section we provide details of the two sets of data used to build our models. We include additional tables constructed from the original data sets. We also give a brief description of the algorithm we use to perform the numerical simulations shown in the paper.

Description of data used for the models

The data we have used for the models were taken from references [8] and [23] of the main text. The fraction of eggs that hatch a given number of days after oviposition are given in Table A1. These data do not include mortality of the whole egg stage, which was found to be 25% for Takano-Lee (TL) data and 12.3% for Evans-Smith (ES) ones. Table A2 gives the fraction of lice that undergo each moult a given number of days after hatching. Mortality is included in these sets of data and consequently the fractions for each moult do not add up to 1. The average mortality of the whole larvar stage is 27% (TL) and 10% (ES). As from the data it is not possible to know how many lice or eggs died each day, we have used in our model a mortality rate of 3% per day (TL) and 0.8% per day (ES) for both the egg and larval stages. These values were chosen so that the average mortality obtained from them is consistent with the average mortality data.

We have assumed that females lay a daily average of 5 eggs, from their fourth day of adulthood until their deaths. For the first three days we have assumed that females lay an average of 0, 2 and 4 eggs, respectively. This corresponds to the rise observed in egg production in both sets of data. Moreover, we have assumed that half of the eggs that hatch give rise to female lice, because no data set was reported to be sex-biased.

Table A1. Fraction of eggs that hatch at a given day after oviposition, with respect to the total of eggs that hatch (i.e. eggs which do not hatch are not taken into account).

Takano-Lee		Evans-Smith	
days	fraction	days	fraction
7	0.299	6	0.144
8	0.285	7	0.174
9	0.211	8	0.298
10	0.127	9	0.305
11	0.078	10	0.079

For the ES data, the fraction of lice that are alive x days after the last moult can be fitted by a Weibull function: $f(x) = \exp(-x/a)^2$, with $a = 20.0$. Fig. A1 shows that the fit is very good for female lice. The TL data are not so detailed and only give average and variance for the mortalities. We have assumed that the functional behaviour should be the same as for ES data and therefore we have simply looked for the value of a which gives the average and variance of the Weibull function closest to the TL data: average mortality 20.2 ± 1.4. The value found is $a = 22.8$ which gives an average of 20.2 ± 2.3. Note that the number of lice for which the TL average was obtained is relatively small: 19 female lice.

A remark is in order regarding TL data. In their paper, the authors give the vital parameters of three *strains* of head lice, collected in three different places: California, Ecuador, and Florida. We have only used the data corresponding to the California strain because it has better statistics (more individuals

Table A2. Fraction of lice that moult at a given day after hatching. Fractions are expressed respect to the total of lice that have undergone the previous moult. Note that for some moults fractions do not add up to 1, reflecting the fact that some lice died before that moult.

	Takano-Lee		Evans-Smith	
	days	fraction	days	fraction
First Moult	3	0.96	4	0.141
	4	0.04	5	0.488
			6	0.308
			7	0.026
Second Moult	5	0.673	8	0.413
	6	0.154	9	0.512
	7	0.02	10	0.050
Third Moult	8	0.5	12	0.305
	9	0.23	13	0.467
			14	0.133

for many life stages) and, more importantly, because the differences between strains are smaller than differences between the California strain data and ES data.

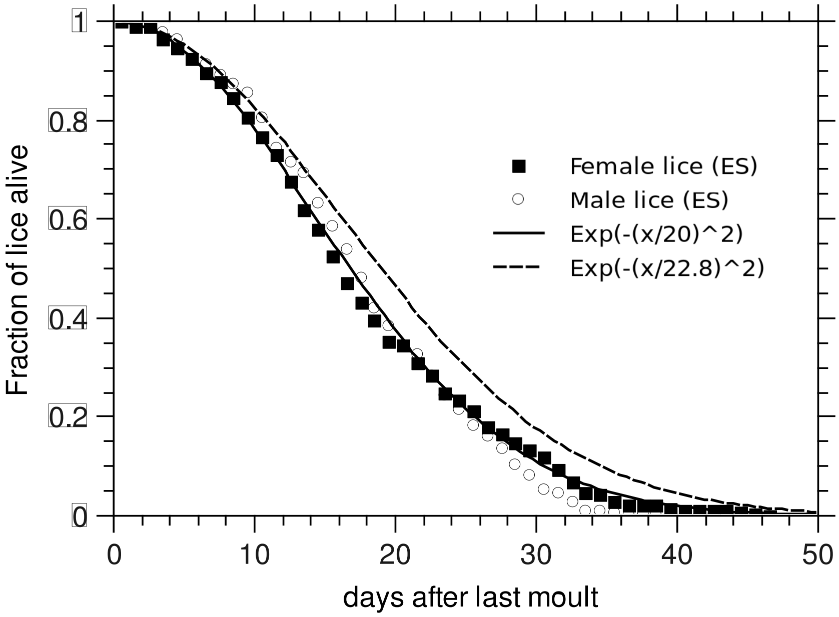

Figure A1. Survival curve of head lice. Fraction of lice alive as a function of the number of days elapsed since the last moult. Symbols correspond to ES data. The full curve is a fit to the curve of female lice mortality. The dotted curve gives the same average and variance of the mortalities in the TL data.

Simulations

As there are many relevant variables that cannot be calculated with the theoretical approach, we have resorted to stochastic simulations. This also allow us to see what happens in single infestations. In the following we give a brief description of the algorithm used.

All simulations start with an adult female louse that has moulted 10 days before. The number of days that she will live is a random number drawn from the corresponding mortality distribution (mentioned in the previous subsection). As, in principle, there is no difference in behaviour between the three larval stages, we group all nymphs in the same class, independently of the number of molts each has undergone. For this reason, we have only used the data concerning the third molt (Table A2), which is the moment when nymphs turn into adults.

In a 'day' of computation the following steps are followed:
1) *grooming*: each adult and nymph is killed with a given probability (.05 in all cases).
2) *nymph and egg mortality*: each nymph and egg is killed with a probability given probability (.008 for ES data and .03 for TL ones).
3) *births*: for each alive female adult lice, a random integer number is drawn to know how many eggs she will lay. This depends on her age:
-adults less than 3 days old lay no eggs.
-3-day olds lay 1, 2, or 3 eggs with probabilities 0.25, 0.5, 0.25, respectively.
-4-day olds lay 3, 4, or 5 eggs with probabilities 0.25, 0.5, 0.25, respectively.
-adults older than 4 days lay 4, 5, or 6 eggs with probabilities 0.25, 0.5, 0.25 respectively.
Each egg is assigned 3 random integer numbers corresponding to the day each of the life stages will begin: hatching, third moult, and death. The hatching day is obtained by randomly choosing a number between 7 and 11 for TL, or between 6 and 10 for ES, with the probabilities given in table A1. For the third moult day we have rescaled the data of Table A2, since it includes mortality, which is already taken into account in step 2 of this algorithm. The rescaled sets of data where used to determine the day of the third moult. For TL data we choose the third moult 8 days after hatching with probability 0.685, and 9 days after hatching with probability 0.315. For ES data, the third moult day is picked as 12, 13 or 14 days after hatching, with probabilities 0.337, 0.516 and 0.147, respectively. The death day has been selected from the distributions mentioned in the previous subsection. The internal clock of each new egg is set to 0.
4) *death*: all adults whose internal clock reaches the established date of death are killed.
5) *contagion*: each alive female adult louse jumps to a randomly chosen head with a probability p_t.
6) *treatment*: each adult and nymph is killed with a probability given by the pediculicidity of the application, and eggs are killed with the corresponding ovicidal probability.
7) *counting*: the number of alive lice in each life stage is counted.
8) *updating*: the internal clock of each alive louse is incremented in one day.

In the oviposition step we have chosen a set of probabilities for the numbers of eggs layed, to add some stochasticity to the process. But we have checked that, given that the average is conserved, results depend only very weakly on these probabilities.

Section B: Figures from ES data

In the main text, most tables and figures are made only with the TL data, to avoid a multiplication of figures and tables. In this section we provide for each of them in the main text its analogue built from ES data. To make the comparison easier, figures and tables have the same number as their analogue in the main text, but preceded by a letter B.

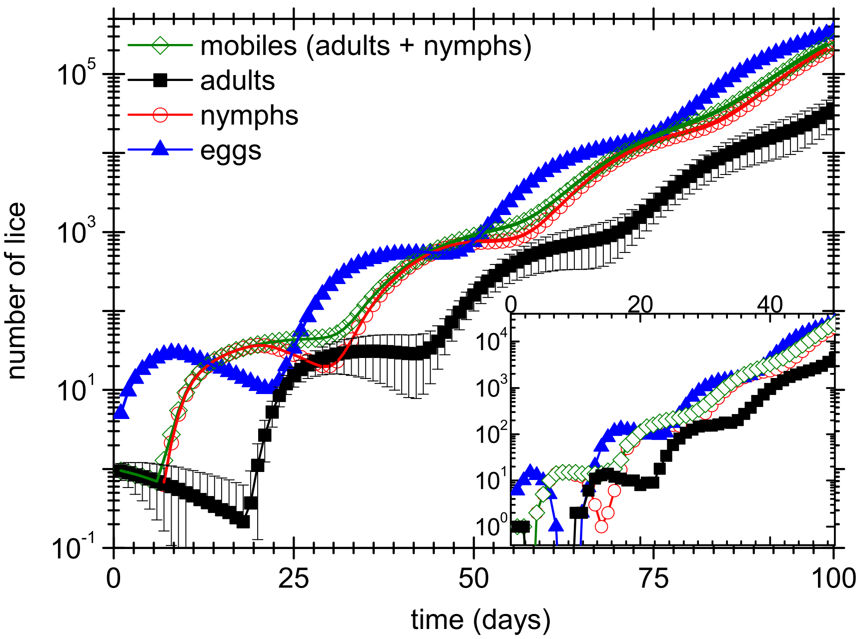

Figure B1. Evolution of lice colonies. Average number of lice of a colony that is started at day 0 by a female that had her last moult 10 days before, as a function of time. Symbols represent averages taken over 1000 populations whereas full lines represent the theoretical predictions. The inset shows the first days of one of these populations. Here, as in the rest of the figures, the error bars represent the standard error of the mean, and data whose error bars are not shown have standard errors lower than symbol size.

Table B3. Result of the average of several variables over 1000 runs of collective infestations for scenario 1 (group of 20 heads with thresholds for treatment start randomly chosen between 10 and 20 mobile lice). In the first column the probability of transfer is indicated. The second column gives the average total daily number of mobile lice present. The third is the prevalence, i.e., the proportion of infested heads. Fourth column indicates the average daily number of lice transferred from one head to another. The fifth and sixth columns give the average and median duration of collective infestations, respectively.

p_t	total mobile	prevalence	total transferred	average duration of infestation	median duration of infestation
0.010	3.67	4.57%	0.095	27.11	24
0.050	4.48	5.78%	0.82	34.22	24
0.075	5.43	6.94%	2.53	46.41	27
0.100	6.97	8.63%	6.60	63.94	33

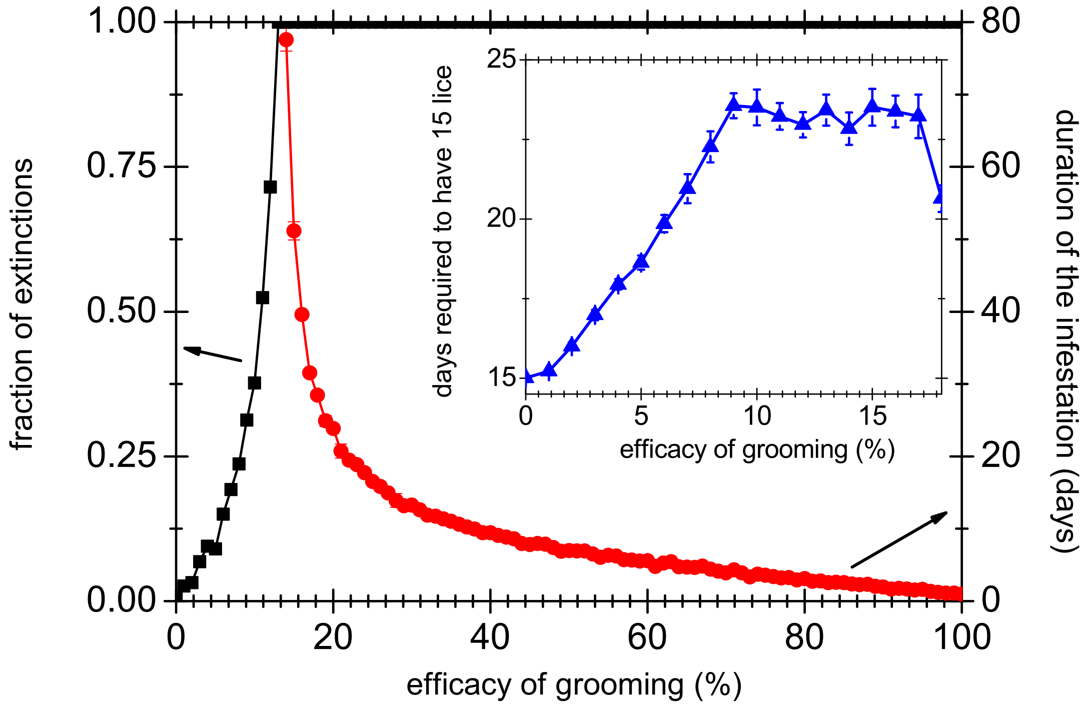

Figure B3. Extinctions and duration of the infestations. Fraction of extinctions (squares), and average duration of the infestation for the extinct populations (circles), as a function of the efficiency of grooming. Averages were taken over 1000 realizations, with a limit time of 500 simulation days (i.e. we only count extinctions happening within the first 500 days). The critical grooming efficacy is defined as the value at which the fraction of extinctions reaches unity. In this plot, this happens for efficacies close to 15%. Inset: Number of days required to reach a population of 15 lice as a function of the efficacy of grooming.

Table B4. Result of the average of several variables over 1000 runs of collective infestations for scenario 2 (almost identical to scenario 1, but with one individual having a treatment threshold of 100 mobile lice). In the first column the probability of transfer is indicated. Second column gives the average total daily number of mobile lice involved. The third is the prevalence, i.e., the proportion of infested heads. Fourth column indicates the average daily number of lice transferred from one head to another. The fifth and sixth columns give the average and median duration of collective infestations, respectively.

p_t	total mobile	prevalence	total transferred	average duration of infestation	median duration of infestation
0.010	22.77	6.55%	1.40	72.59	73
0.050	25.52	14.35%	12.53	112.13	107
0.075	29.36	19.65%	30.89	161.02	139
0.100	35.54	25.8%	71.80	231.59	182

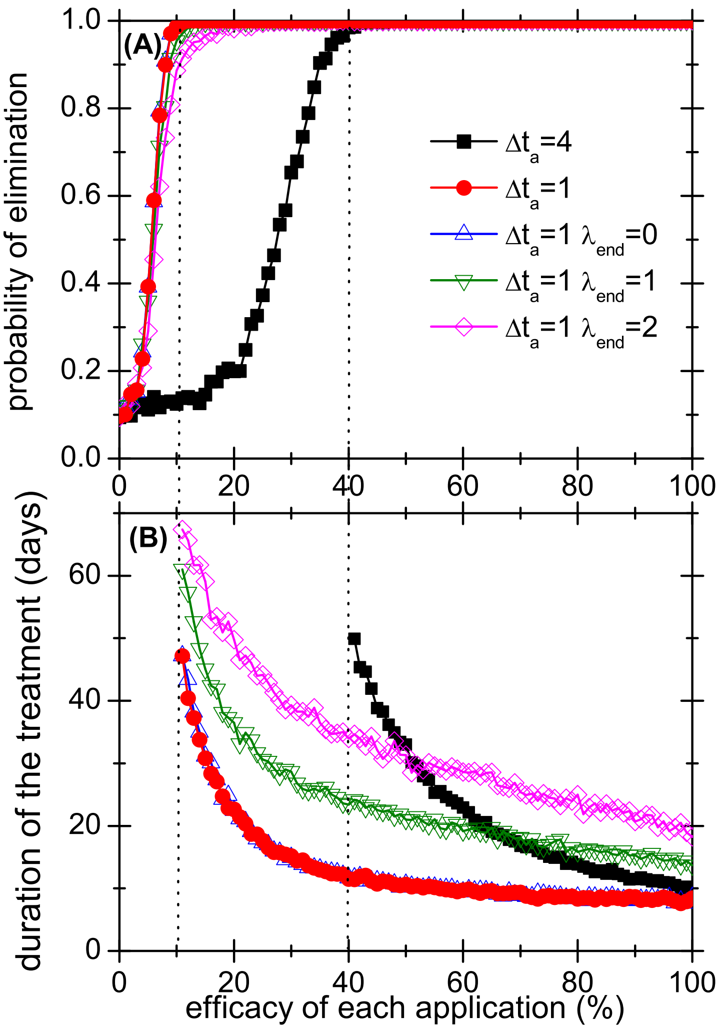

Figure B4. Results of applying different treatments to cure a head lice infestation. The upper panel shows the probability that the infestation is cured (i.e. that all head lice and eggs are eliminated) and the lower panel shows the duration of the treatment, when it is successful. Both variables are plotted as functions of the fraction of lice that are eliminated by each application of the treatment for a fixed ovicidity of 10%. The limit time in our simulations to allow for the extinction of the colony was 500 days of simulation time. Dotted vertical lines indicate the critical efficacy of the treatments.

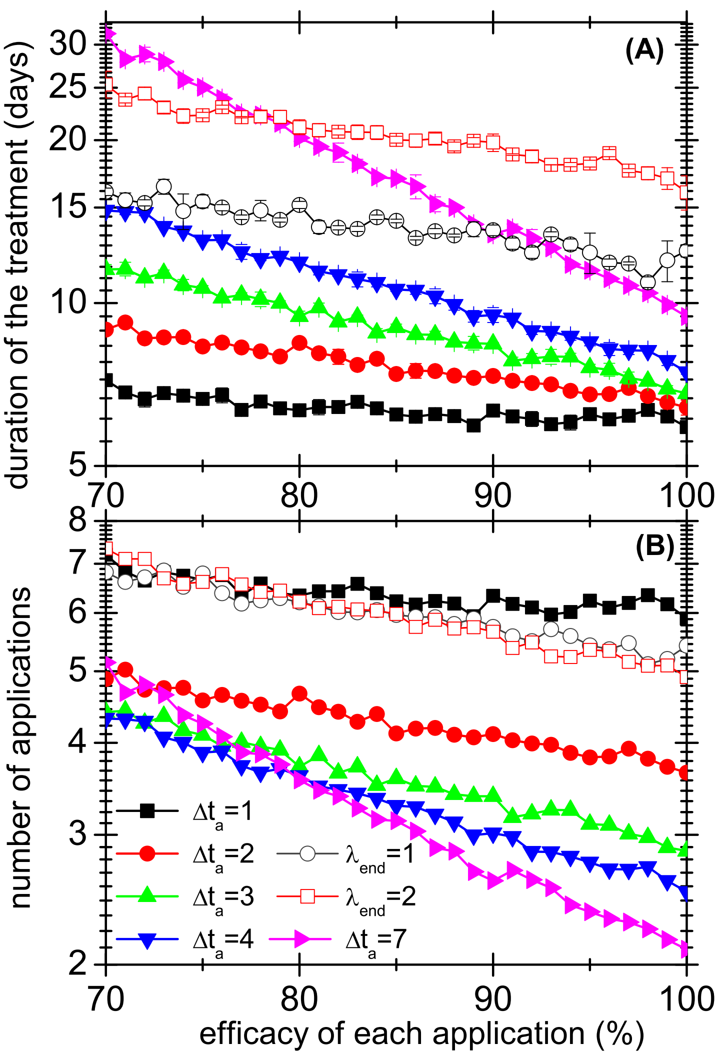

Figure B5. Average durations and number of necessary applications of different treatments. Average durations are shown in panel A and the number of necessary applications to cure the infestation are shown in panel B. Results correspond to several treatments, when they are successful, and are shown as a function of the fraction of lice eliminated by each application. Full symbols indicate systematic treatments, and Δt_a gives the number of days between applications. Empty symbols correspond to a daily application ($\Delta t_a = 1$) of non systematic treatments which are stopped when the mobile lice remaining in the population are less of equal than λ_{end}.

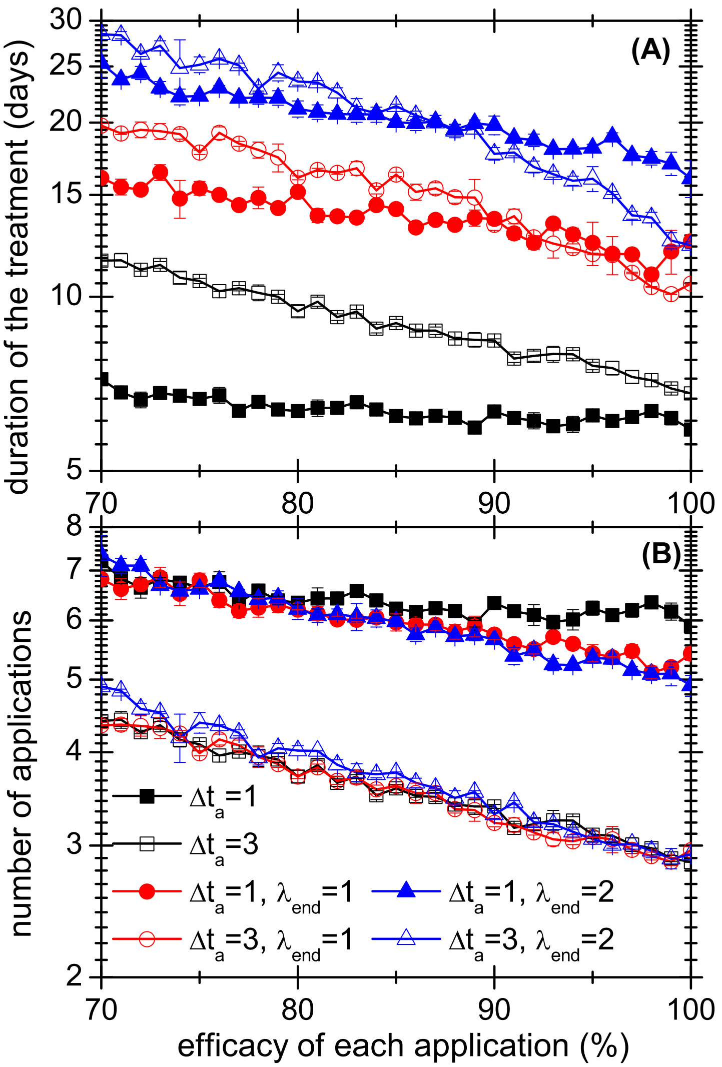

Figure B6. Average durations and number of necessary applications of different treatments. Average durations are shown in panel A and the number of necessary applications to cure the infestation are shown in panel B. Results correspond to several treatments, when they are successful, and are shown as a function of the fraction of lice eliminated by each application. Squared symbols are systematic treatments, whereas the rest are non systematic ones. Full symbols correspond to daily applications that are stopped when less than λ_{end} mobile lice remain in the population. Empty symbols correspond to an application every 3 days.

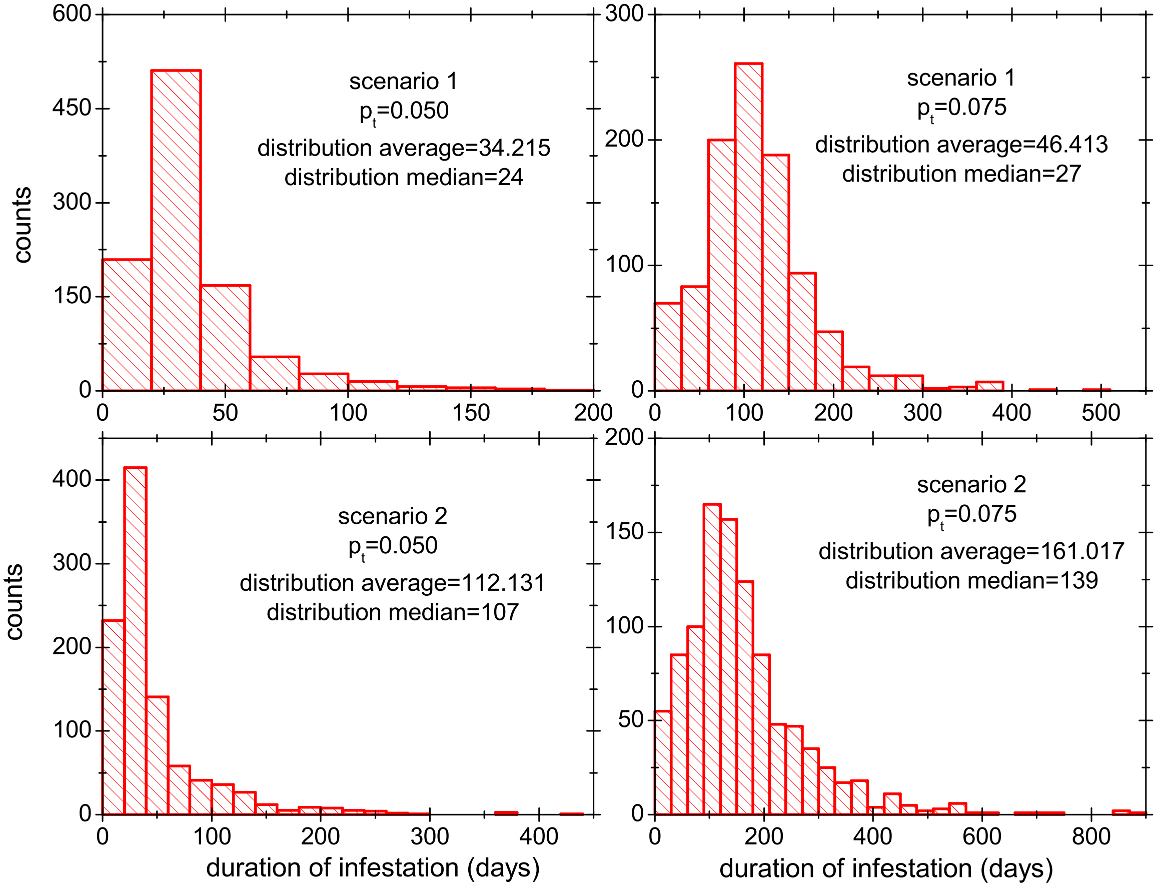

Figure B7. Histograms of the duration of the collective infestations. The histograms were built from 1000 runs of scenario 1 (upper panels) and scenario 2 (lower panels) for two values of the probability of transfer: $p_t = 0.05$ (left panels) and $p_t = 0.075$ (right panels).

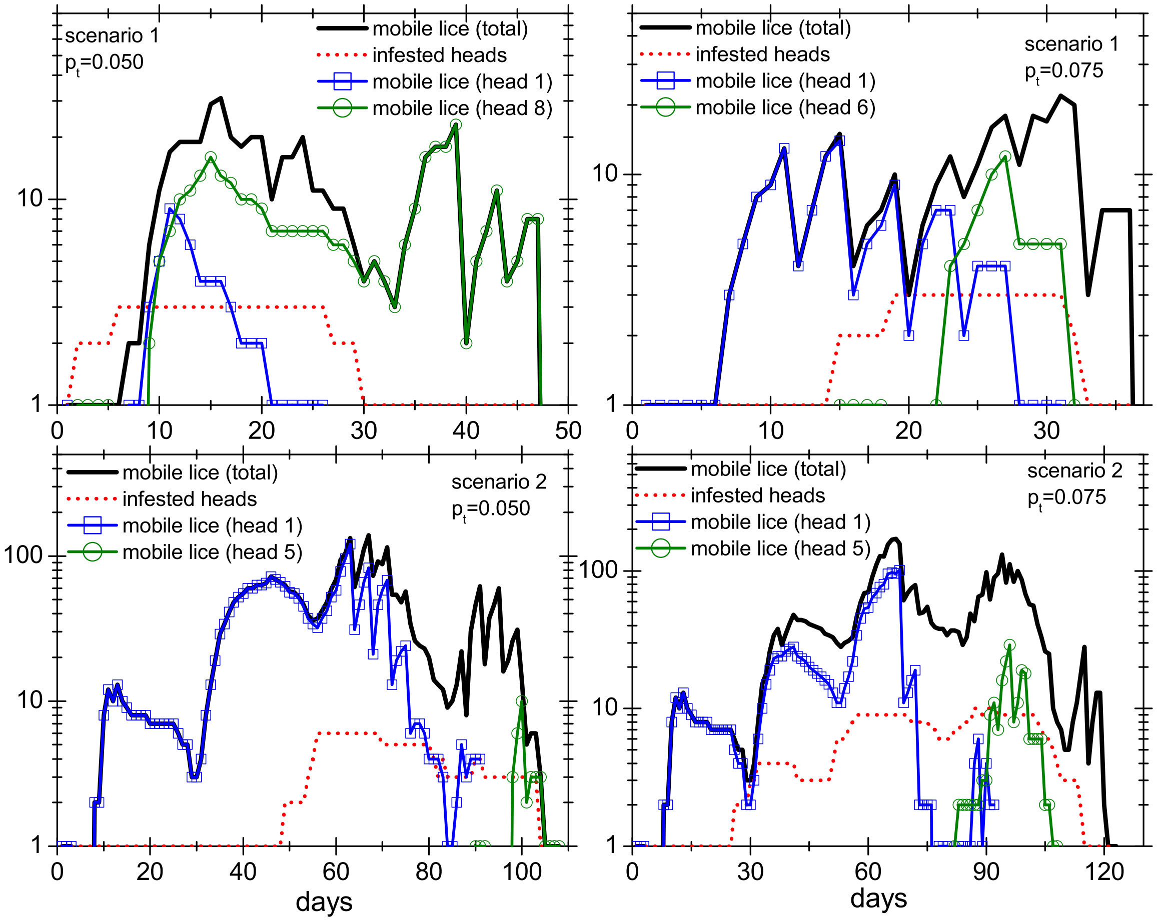

Figure B8. Time evolution of 4 different collective infestations. Behavior of the colonies in scenario 1 (upper panels) and scenario 2 (lower panels) and for two values of the probability of transfer: $p_t = 0.05$ (left panels) and $p_t = 0.075$ (right panels). In all panels the total number of mobile lice as well as the number of infested heads is shown. For the sake of clarity, we only show the evolution of the infestation of two heads. Head 1 is the one where the first female louse appears, whereas the other is randomly chosen. Besides, for scenario 2 head 1 is also the superspreader head.

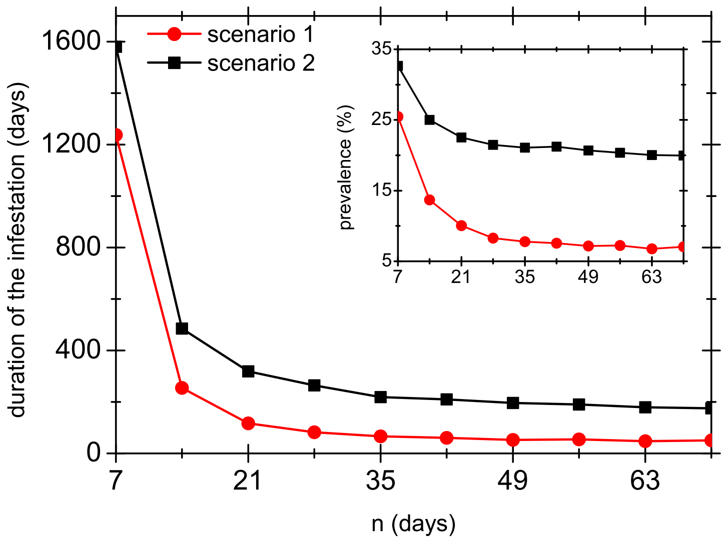

Figure B9. Effect of the inclusion of external lice. Average duration of infestation for a group of 20 heads in which an external female adult louse is introduced every n days. Transmission probability is $p_t = 0.075$ and the treatment used consists of applications of 80% efficacy every 4 days. Symbols represent averages over 1000 simulations (lines are guides to the eye). The inset shows the prevalence for the same situation, as a function of n.

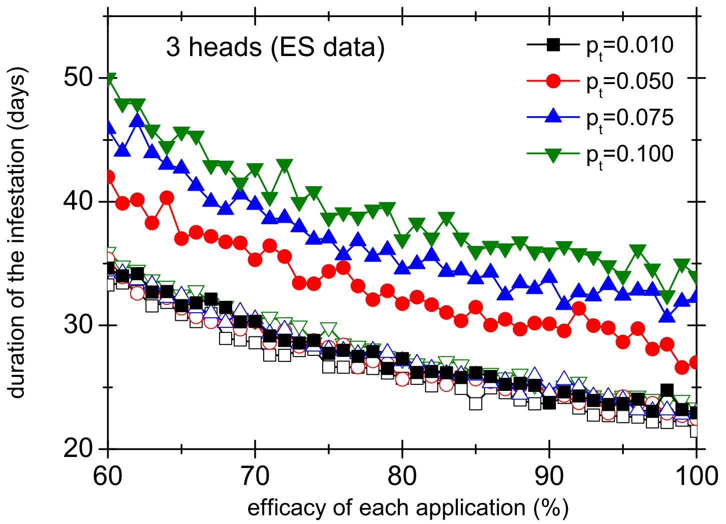

Figure B10. Performance of different treatments in a group of 3 heads. Duration of the infestation as a function of the efficacy of each application for a systematic treatment applied every 4 days, implemented in a synchronized (open symbols) and unsynchronized way (full symbols). Different curves correspond to different values of the transmission probability p_t. For the unsynchronized case we have used scenario 1.

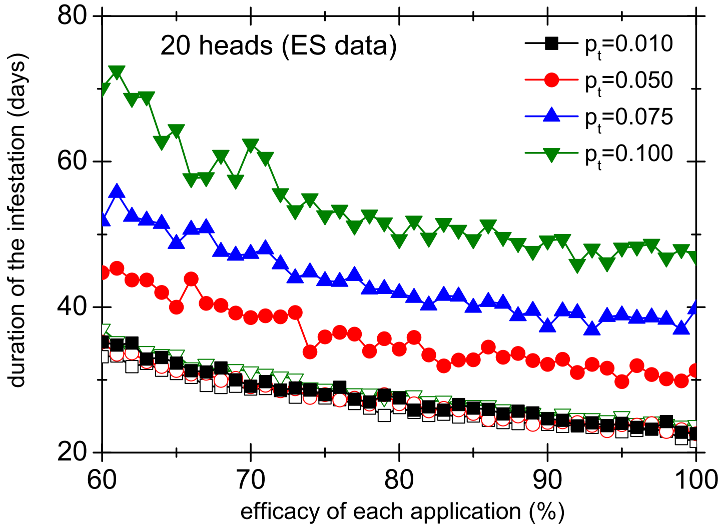

Figure B11. Performance of different treatments in a group of 20 heads. Duration of the infestation as a function of the efficacy of each application for a systematic treatment applied every 4 days, implemented in a synchronized (open symbols) and unsynchronized way (full symbols). Different curves correspond to different values of the transmission probability p_t. For the unsynchronized case we have used scenario 1.

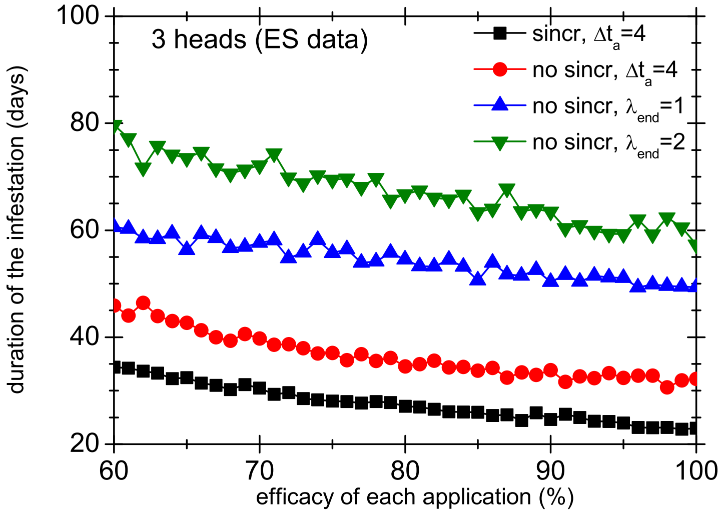

Figure B12. Effect of treatment systematicity on a group of 3 heads. Average duration over 1000 realizations of 4 different treatments in group of 3 heads, as a function of the efficacy of each application. Squares and circles correspond to systematic treatments, whereas triangles represent non-systematic ones.

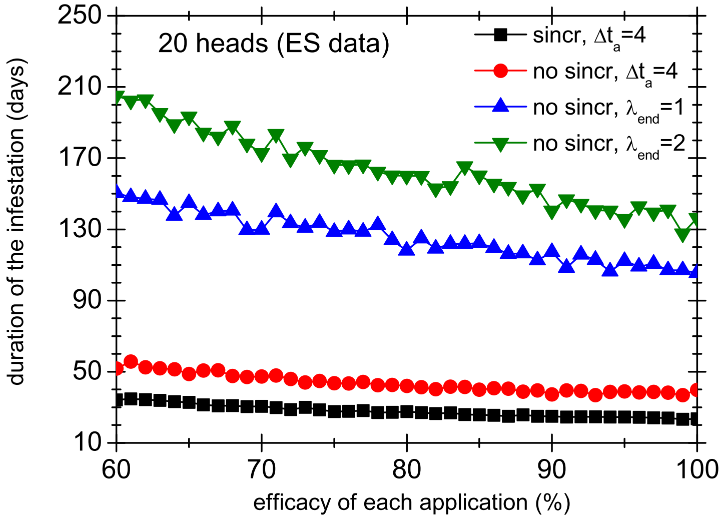

Figure B13. Effect of treatment systematicity on a group of 20 heads. Average duration over 1000 realizations of 4 different treatments in group of 3 heads, as a function of the efficacy of each application. Squares and circles correspond to systematic treatments, whereas triangles represent non-systematic ones.
